# Prevalence of self-reported complications associated with intermittent catheterization in wheelchair athletes with spinal cord injury

**DOI:** 10.1038/s41393-020-00565-6

**Published:** 2020-10-13

**Authors:** Matthias Walter, Ian Ruiz, Jordan W. Squair, Luis A. S. Rios, Marcio A. Averbeck, Andrei V. Krassioukov

**Affiliations:** 1 International Collaboration on Repair Discoveries (ICORD), Faculty of Medicine, University of British Columbia (UBC), Vancouver, British Columbia (BC) Canada; 2 Department of Urology, University Hospital Basel, University of Basel, Basel, Switzerland; 3 Department of Urology, IAMSPE Hospital, São Paulo, Brazil; 4 Videourodynamic Unit, Albert Einstein Hospital, São Paulo, Brazil; 5 Department of Urology, Moinhos de Vento Hospital, Porto Alegre, Brazil; 6 Division of Physical Medicine and Rehabilitation, Faculty of Medicine, UBC, Vancouver, BC Canada; 7 G.F. Strong Rehabilitation Centre, Vancouver, BC Canada

**Keywords:** Spinal cord diseases, Neurogenic bladder

## Abstract

**Study design:**

Cross-sectional study.

**Objectives:**

To identify the prevalence of complications associated with intermittent catheterization in wheelchair athletes with spinal cord injury (SCI).

**Setting:**

International and national sporting events.

**Methods:**

A total 130 competitive wheelchair athletes living with SCI completed a self-reported questionnaire during international or national sporting events. The questionnaire collected information regarding demographics, injury characteristics, method of bladder emptying, and complications related to intermittent catheterization.

**Results:**

Overall, 84% (109/130) of wheelchair athletes used intermittent catheterization. Within this group, 77% of athletes (84/109) experienced at least one complication associated with intermittent catheterization. Twenty-seven percent (29/109) sustained urethral injuries and 63% (69/109) had at least one episode of urinary tract infection during the last 12 months. Almost one-fourth of male athletes (22/95, 23%) had a history of inflammation / infection of genital organs associated with intermittent catheterization.

**Conclusions:**

Here we report a high prevalence of self-reported complications associated with intermittent catheterization in wheelchair athletes with SCI. Considering their potential impact on lower urinary tract function, athletic performance, and health, further studies are needed to assess the role of preventative strategies to reduce complications related to intermittent catheterization in wheelchair athletes with SCI.

**Sponsorship:**

Coloplast Brazil and Instituto Lado a Lado pela Vida (a nongovernmental, nonprofit organization based in São Paulo) and Wellspect provided funding for this study.

## Introduction

Following spinal cord injury (SCI), the vast majority of individuals suffer from urinary incontinence and/or the inability to void spontaneously as a consequence of impaired supraspinal control of the lower urinary tract (LUT) [1]. As this negatively impacts quality of life, it comes as no surprise that individuals living with SCI rank the management of adult neurogenic LUT dysfunction (ANLUTD) as a top health priority [2, 3]. For decades, both indwelling and intermittent catheterization (IC) practices have been used to facilitate bladder emptying in individuals living with SCI [4]. Unfortunately, indwelling catheters (suprapubic or urethral) are associated with a high risk of urinary tract infection (UTI) [5] and bladder cancer [6]. In contrast, IC has been shown to better preserve LUT function [7], while also reducing the risk of upper and LUT complications [8–10]. Thus, IC is now considered the gold standard for bladder emptying in individuals following SCI with sufficient manual dexterity [11, 12].

Despite the development of improvements in urethral catheter properties (e.g., hydrophilic coating), complications such as UTIs and urethral injuries are still highly prevalent [13] and pose a negative impact on quality of life and continue to be a tremendous economic burden on healthcare systems [14, 15]. While single-use catheters have been shown to be cost-effective [16], insufficient coverage from healthcare insurance providers continues to be a significant concern, likely contributing to their reuse by individuals living with SCI. Moreover, reuse of single-use catheters has been associated with an increased risk of UTI [17].

In contrast to the entire SCI population, athletes with SCI have received far less attention in the past. However, due to the growing popularity of Paralympics, research groups have shifted their attention to this subpopulation. The International Paralympic Committee (IPC) is committed to ensure athlete health and encourages all initiatives and measures to minimize the risk of injury and illness in Paralympic athletes. Though, wheelchair athletes have unique pre-existing medical conditions that predispose them to increased risk of illness [18]. However, little is known with respect to morbidity as it relates to an athlete’s LUT management. While high levels of physical activity have been shown to improve fitness [19] and reduce cardiometabolic disease risk factors [20] in individuals with SCI, complications associated with IC, especially recurrent UTIs, are detriment to an individual’s health and may possibly lead to frequent clinic visits and hospitalizations. As a consequence, sport performance may also be negatively impacted due to periods of inactivity, interfering with training and performance in athletes with SCI [17]. As evident from the study by Derman et al. the genitourinary illnesses in the Paralympic population are more prevalent than in able-bodied athletes [21].

As management of ANLUTD continues to be an ongoing concern for individuals with SCI with limited healthcare coverage owing to the lack of data in this population, we aimed to identify the prevalence of self-reported complications associated with IC among a physically more active healthy subpopulation of individuals with SCI (i.e., wheelchair athletes).

## Methods

### Study participants

Competitive wheelchair athletes with SCI completed a self-reported questionnaire during international or national sporting events (2015 ParaPan American Games, Toronto, Canada; The 2016 Summer Paralympics, Rio de Janeiro, Brazil; the 2017 Prairie League Tournament, Calgary, Canada; and the 2017 Invitational Wheelchair Rugby Tournament, Vancouver, Canada). Inclusion criteria comprised wheelchair athletes with SCI of both sexes between the age of 18 and 55 years. There were no additional exclusion criteria.

### Study design

This cross-sectional study was conducted using a self-reported questionnaire. The Delphi method was used to develop this questionnaire with a recruited panel of experts, which included individuals with SCI, clinicians, and scientist with expertise in SCI from the International collaboration on repair discoveries and Vancouver coastal health research institute located at the Blusson spinal cord centre in Vancouver, Canada. The questionnaire (supplementary file 1) included 30 multiple choices questions and comprised the following four categories:

- Demographics and severity of SCI according to the International Standards for Neurological Classification of SCI (ISNCSCI) [22]
- Questions from the ‘Autonomic Standards Assessment Form’ - International Standards to document remaining Autonomic Function after SCI (ISAFSCI) [23]
- History of catheterization techniques and associated complications, such as urethral injuries and UTI
- History of inflammation/infection of genital organs associated with IC in male individuals

The original English questionnaire was used to interview wheelchair athletes with SCI in Calgary, Toronto, and Vancouver. A translated Portuguese version was utilized to interview Brazilian wheelchair athletes with SCI at the 2016 Summer Paralympics in Rio de Janeiro, Brazil.

### Outcome objectives

The two primary outcome objectives of this study were the prevalence of urethral injuries and UTIs associated with IC among wheelchair athletes with SCI. The secondary outcome objectives were the number of wheelchair athletes with SCI reporting difficulties introducing the catheter, pain during catheterization, and the presence of blood on the catheter after catheterization as well as the prevalence of inflammation/infection of male genital organs associated with IC. Tertiary outcome objective was the distribution of IC characteristics, i.e., type of catheter (hydrophilic or nonhydrophilic), use of catheter (single-use or reuse), use of lubrication, frequency of catheterizations per day, size of catheters, and shape of catheter tip (straight or curved/bent).

### Statistical analysis

Data are presented as raw values and percentages. Furthermore, data were assessed for normal distribution using the Kolmogorov-Smirnov test. Thus, results for age, time after SCI, number of UTIs within the last 12 months, antibiotic treatments for UTI within the last 12 months, years using IC, and frequency of catheterization per day are presented as median with interquartile range (IQR) and range (i.e., min - max). The prevalence of a complication associated with IC, such as UTI or urethral injury, is defined as the number of participants reporting such a complication within the entire cohort of wheelchair athletes performing IC in this cross-sectional study. Statistical analyses were conducted using R Statistical Software Version 3.6.0 for Mac Os.

## Results

### Participant Characteristics

A total of 130 competitive wheelchair athletes with SCI (i.e., Toronto [*n* = 27]; Rio de Janeiro [*n* = 14]; Calgary [*n* = 38]; and Vancouver [*n* = 51]) including 18 females (14%) completed the questionnaire once (i.e., no duplicate reports). None of the eligible wheelchair athletes declined participation in this study. Table 1 summarizes baseline demographics and SCI characteristics. Median age and time after SCI were 34 (IQR 28–41, range 18–55) and 12 (IQR 9 to 19, range 2–39) years respectively. The majority of our participants had a cervical (88%, 115/130) and complete (79%, 103/130) injury. Although there was a heterogeneity of the athletes’ choice for the method of bladder emptying, the majority used IC (84%, 109/130). Within this subgroup of athletes, IC was utilized as the primary (93/109) methods of bladder emptying or as a secondary (15/109) or tertiary (1/109) option in addition to others methods. Only athletes using IC – previously or currently – were included for further analyses. Among athletes using IC, awareness to empty the bladder and ability to prevent urinary leakage were predominantly impaired (59%, 64/109 and 38%, 41/109) or lost (24% 26/109 and 47%, 51/109) (Table 2). Despite this fact, only 65% (71/109) of athletes were using any form of treatment to aid their ANLUTD (e.g., neurogenic urinary incontinence or NDO). Of those, the majority reported antimuscarinic medication (65%, 46/71) as their primary treatment option. Other athletes reported intradetrusor onabotulinumtoxinA injections (20%, 14/71), use of antibiotic medication as UTI prevention (4%, 3/71), or other (11%, 8/71).

**Table 1:** Baseline demographics and self-reported spinal cord injury characteristics.

Characteristics	All participants (*n* = 130)
Age in years	34 (28–41, 18–55)
Time after SCI in years	12 (9–19, 2–39)
Sex	
Female vs. male	18 (14%) vs. 112 (86%)
Nationality	
Canadian	61 (47%)
United States of America	32 (25%)
Brazilian	24 (18%)
Chile	7 (5%)
Japanese	5 (4%)
Columbia	1 (1%)
Completeness of lesion	
AIS A	55 (42%)
AIS B	48 (37%)
AIS C	11 (8%)
AIS D	5 (4%)
Brown-Sequard-Syndrome	1 (1%)
Unknown (all incomplete)	10 (8%)
Type of plegia	
Tetraplegic vs. paraplegic	116 (89%) vs. 13 (10%)
Unknown	1 (1%)
Level of lesion	
Cervical	115 (88%)
Thoracic	11 (8%)
Lumbar	1 (1%)
Sacral	2 (2%)
Unknown	1 (1%)
Primary method of bladder emptying	
Intermittent catheterization (transurethral)	93 (72%)
Intermittent catheterization (stoma)	3 (2%)
Suprapubic catheter	11 (8%)
Transurethral catheter	5 (4%)
Credé maneuver	2 (2%)
Spontaneous voiding	8 (6%)
Other (including condom catheter)	8 (6%)

Age and time after SCI are presented as median with IQR and range.

*AIS* American Spinal Injury Association Impairment Scale, *IQR* interquartile range, *SCI* spinal cord injury.

**Table 2:** Awareness to empty the bladder and ability to prevent urinary leakage in wheelchair athletes with SCI performing intermittent catheterization.

Characteristics	Participants performing intermittent catheterization (*n* = 109)
Awareness to empty the bladder	
Normal	7 (6%)
Reduced/impaired	64 (59%)
Complete loss	26 (24%)
Unknown	12 (11%)
Ability to prevent urinary leakage	
Normal	5 (4%)
Reduced/impaired	41 (38%)
Complete loss	51 (47%)
Unknown	12 (11%)

### Complications associated with intermittent catheterization

**Fig. 1:**
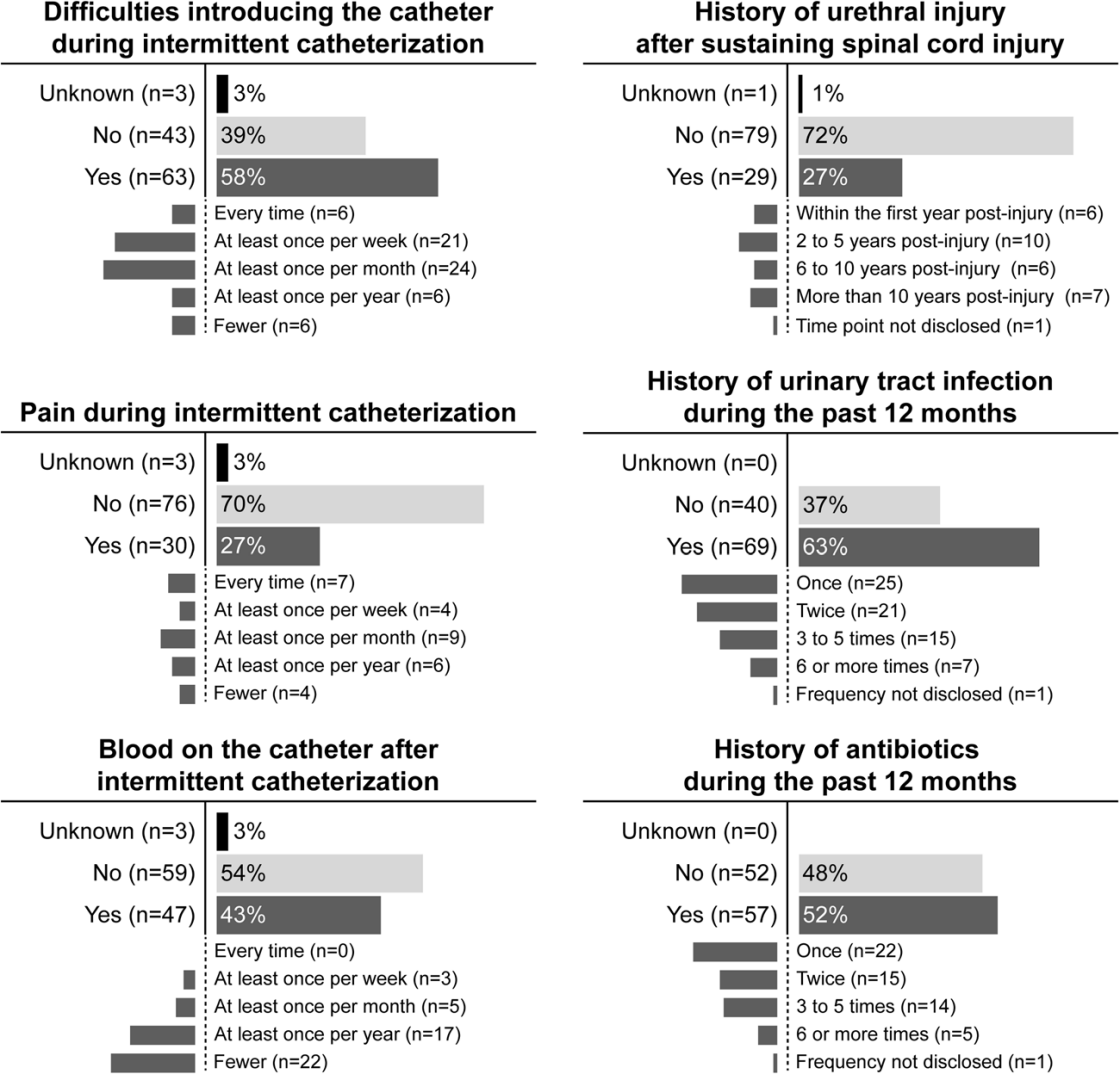
Complications associated with intermittent catheterization. Summary of distribution and frequency of self-reported complications  in wheelchair athletes performing intermittent catheterization (*n* = 109).

Overall, 84 athletes (77%) reported to have experienced at least one complication associated with IC since sustaining their SCI (see Fig. 1). At least one episode of UTI during the last 12 months was reported by 63% of athletes (69/109). The median number of self-reported UTIs per year was 1 (IQR 0–2, range 0–12). More than half of the athletes (52%, 57/109) underwent at least one course of antibiotic treatment for UTI during the last 12 months. The median number of antibiotic treatments for UTI per year was 1 (IQR 0–2, range 0–11).

More than one-fourth (27%, 29/109) experienced at least one urethral injury as a direct consequence of IC post-SCI (i.e., as a consequence of self-catheterization: 72%, 21/29 or catheterization through others: 14%, 4/29; following both, self-catheterization and catheterization through others: 7%, 2/29; or due either self-catheterization or catheterization through others: 7%, 2/29). Ninety percent (26/29) of all urethral injuries in our cohort were reported by individuals with a cervical SCI. The remaining urethral injuries were reported by athletes with a thoracic (7%, 2/29) or lumbosacral (3%, 1/29) SCI.

**Fig. 2:**
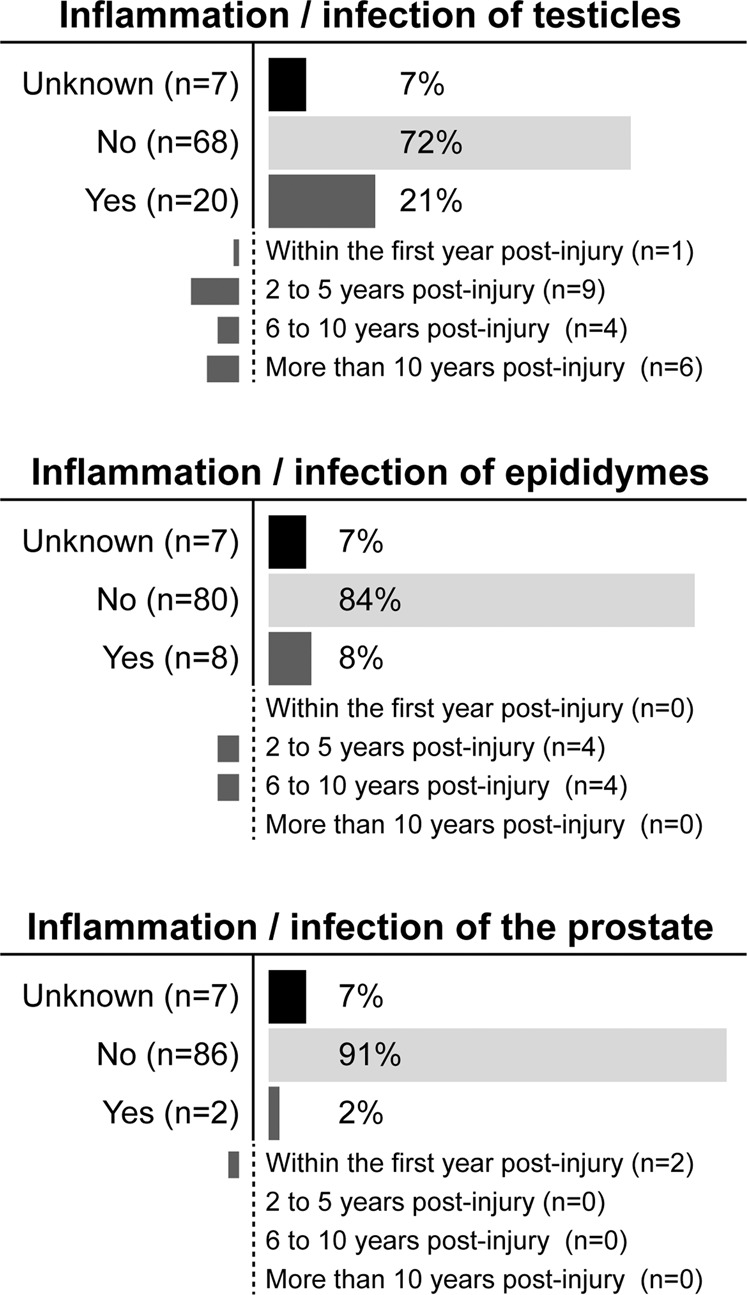
Inflammation/infection of male genital organs. Distribution of self-reported inflammation/infection of genital organs in male athletes performing intermittent catheterization (*n* = 95).

The majority of athletes (58%, 63/109) reported that they experienced difficulties introducing the catheter into the urethra. Furthermore, athletes reported pain during IC (28%, 30/109) or noticed blood on the catheter after IC (43%, 47/109). In the subgroup of male wheelchair athletes (95/109), 23% of participants (22/95) reported at least one episode of inflammation/infection of genital organs (Fig. 2).

### Characteristics of intermittent catheterization

**Fig. 3:**
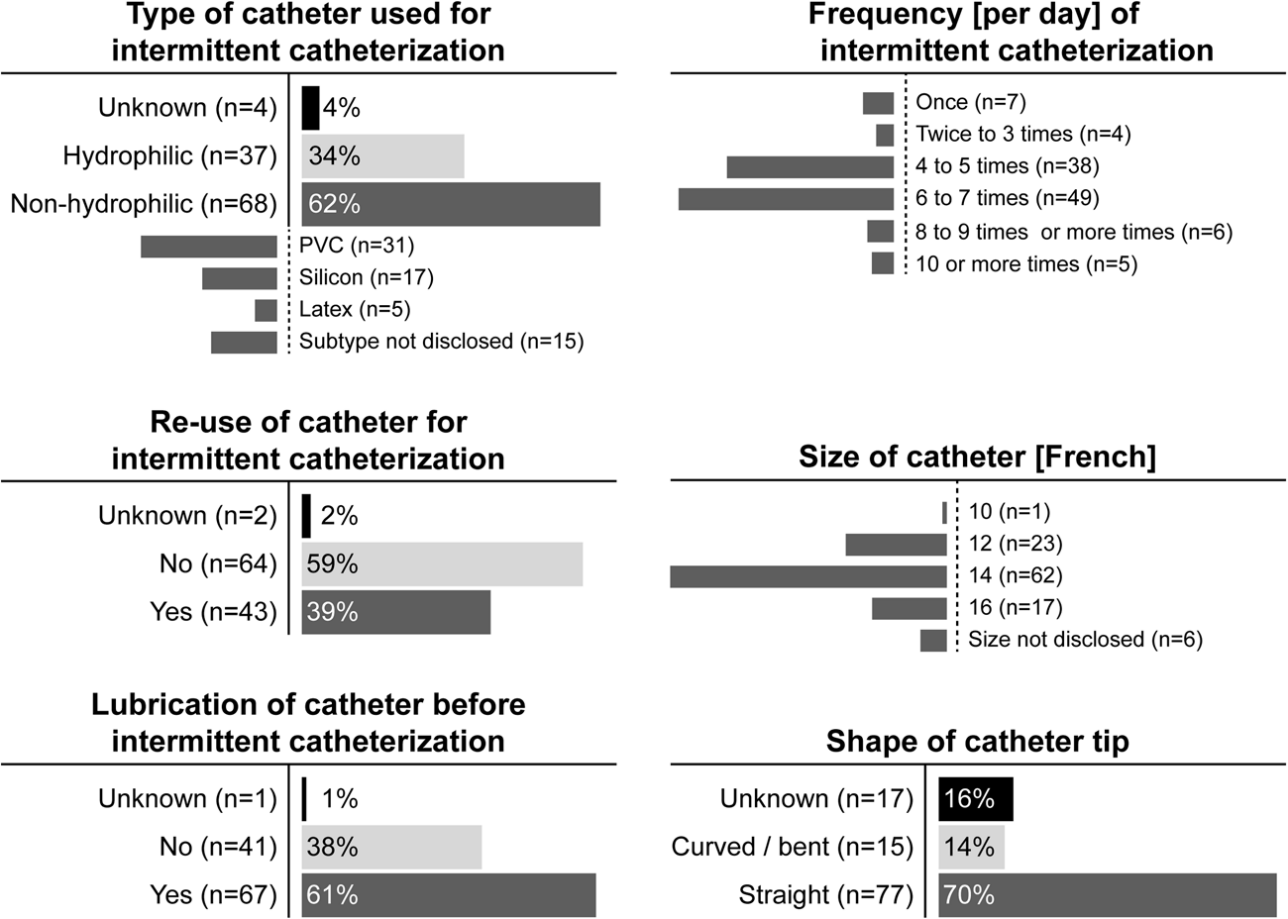
Characteristics of intermittent catheterization. Distribution of key characteristics of intermittent catheterization in wheelchair athletes with SCI (*n* = 109).

The most common characteristics of IC (Fig. 3) in this population were the reported use of nonhydrophilic catheters (62%, 68/109), single-use of catheters (59%, 64/109), lubrication (61%, 67/109), a frequency of 6–7 catheterizations per day (45%, 49/109), size 14 Fr catheters (57%, 62/109) and straight tipped catheters (70%, 77/109). The median duration of performing IC was 10 years (IQR 6–15, range 1–28). The median frequency of catheterizations per day was 5 (IQR 4.5–6, range 1–10).

## Discussion

Although IC has been proposed as the preferred management of ANLUTD whenever sufficient manual dexterity is preserved [7, 8, 11], the associated prevalence / incidence of UTI and urethral injury continues to place a significant economic burden on both the patient and the healthcare system [14, 15]. In our cross-sectional study in 109 wheelchair athletes living with SCI and performing IC, almost 80% reported at least one complication associated with IC post-SCI (e.g., urethral injury in 27% and at least one episode of UTI during the last 12 months in 63%). Furthermore, 23% of male athletes reported a history of inflammation / infection to genital organs post-SCI.

A study by Biering-Sorensen et al. found that 81% (62/77) of their study population had at least one episode of UTI over the course of the first five years following SCI [24]. In another study by Bakke et al. the prevalence of UTIs associated with IC was found to be 76% (307/407) over the course of one year [25]. In line with this, the present study reports a similar rate of UTIs (63%) over the course of the previous 12 months. With each UTI, there is a known risk for the development of upper urinary tract dysfunction as well as autonomic dysreflexia in individuals with SCI at T6 or above, both of which carry serious implications in terms of morbidity and mortality. In particular, autonomic dysreflexia is a well-known risk factor to perpetuate end organ dysfunction [26] as well as to be a significant economic burden to the healthcare system [27]. These sequelae of UTIs on patients’ health and the healthcare system warrant further investigation to address these ongoing concerns.

Seventy-seven percent of our study participants experienced complications with IC including difficulty introducing the catheter into the urethra, pain during catheterization, and noticing blood on the catheter after catheterization. Although these are all potential indicators for an underlying urethral injury as well as risk factors for the development of UTIs, only 27% of our wheelchair athletes reported to have experienced a urethral injury associated with IC following SCI. The onset of urethral injuries in the present study was balanced across the different time periods (i.e., within the first-year, 2–5 years, 6–10 years or thereafter). Cornejo-Dávila et al. [28] reported a 4.2% rate of IC-related urethral strictures in cohort of 333 individuals with SCI. However, since urethral strictures only were observed in men, the sex-adjusted rate of IC-related urethral strictures is 5.6% (i.e., 14/250 men). Wyndaele and Maes [29] reported a 9% rate of urethral strictures related to IC in a cohort of 75 individuals (i.e., 44%, 33/75 males) with SCI and a maximum follow-up of 12 years. In a study by Waller et al. [30] comprising 30 individuals (i.e., 87%, 26/30 males) with SCI and a mean duration of IC of 7 years, a 13% rate of urethral strictures related to IC was reported. Perrouin-Verbe et al. [31] reported on three different groups of individuals performing IC. Their biggest group of 159 individuals (i.e., 71%, 113/159 males) performing IC during the acute phase following SCI had a 5.3% rate of IC-related urethral strictures in the male cohort with a delay of 2.5 years. Their second biggest group (i.e., 21 males who had performed IC for more than 5 years with a mean follow-up of 9.5 years) reported a 19% rate of IC-related urethral strictures with a delay of 4.5 years. Although the prevalence of IC-related urethral injuries in the present study is higher compared to the previous literature, one has to consider that no information on formal testing, such as urethrocystoscopy or retrograde urethrography, is available for confirmation. Therefore, our cohort’s prevalence of urethral injuries could be lower after all. However, the vast majority (~90%, 26/29) of urethral injuries in this present study was reported by individuals with a cervical SCI. Since an SCI at the cervical level may impose a higher risk for IC-related complications due to various reasons (e.g., impaired dexterity) and given the high number of individuals with a cervical injury (90%, 94/109) in our cohort, a potential bias toward a higher prevalence of IC-related complications cannot be ruled out.

As individuals with SCI will continue to rely on IC for decades to come, continued education remains a primary target to address and to attempt to reduce complications associated with IC (e.g., UTIs and urethral injuries). This is an important point, considering that the European Association of Urology Nurses (EAUN, https://nurses.uroweb.org/guideline/catheterisation-urethral-intermittent-in-adults/) has identified poor education as a risk factor for UTIs in patients relying on IC (level of evidence 2b). One target would be to continuously update the SCI population on the latest advances in both catheter types and properties as well as catheterization techniques. Behavior modification may be another target as previous evidence suggests increased rates of UTIs in individuals who reuse their single-use catheters [17].

In our study, more than 40% of participants reused their catheters, exposing them to an increased risk for UTIs and their sequelae. This number of individuals with SCI reusing catheters for IC is similar to studies (35-56%) assessed in the 2014 review by Hakansson [32]. However, our study was neither intended nor powered to assess whether catheter reuse was associated with a higher risk of recurrent UTI and other complications. While single-use catheters have a known significant economic burden associated with their use [33, 34], further research is warranted to identify what other potential educational and/or social factors may be contributing to individuals choosing higher risk healthcare practices over safer ones as well as to provide innovative strategies to reduce IC-related complications in this population. The impact of managing the LUT and IC-related complications is also crucial for Paralympic athletes as genitourinary illnesses are prevalent in this cohort during competitions, which could negatively impact their performance [21].

Currently, there is no consensus on whether the use of hydrophilic (vs. nonhydrophilic) catheters reduces the risk of complications secondary to IC. One study by Cardenas et al. [35] found that using hydrophilic catheters significantly delayed the time to the first symptomatic UTI (i.e., corresponding to a 33% decrease in the daily risk of developing the first symptomatic UTI), which suggests a potential clinical benefit compared to nonhydrophilic catheters. While the majority of our participants (62%) used nonhydrophilic catheters, which is comparable to the reports (50–74%) in the 2014 review by Hakansson [32], our study was neither designed nor sufficiently powered to draw conclusions with respect to complications associated with IC.

Current knowledge on LUT management and its complications in Paralympic athletes with SCI is still scarce. Despite inherent limitations (e.g., the nature of a cross-sectional assessment and the fact that information on UTIs, urethral injury and use of UTI-related antibiotic medication are self-reported only, i.e., without details on specific UTI symptoms and antibiotic resistance as well as objective verification of urethral injuries), our study adds new insights on complications associated with IC in this particular subcohort of individuals with SCI, which may guide further research initiatives in the future.

Since the vast majority of currently available studies are based on insufficient or outdated definitions of UTI, their real-life impact (i.e., prevalence/incidence and severity of UTI) must be considered with great caution. Considering the lack of studies using an adequate catheter-associated UTI definition (e.g., Infectious Diseases Society of America 2009) [36], there is a high demand of well-designed randomized controlled trials. These studies will be able to provide evidence on the impact of reuse vs. single-use catheters and coated vs. uncoated catheters on UTIs as well as to determine predictors of UTIs and urethral injury for individuals performing IC.

## Conclusion

Although IC is the preferred method for bladder emptying in individuals with SCI and preserved hand function, our work revealed a high overall prevalence of self-reported complications associated with IC in wheelchair athletes. Considering their potential impact on LUT function, and in particular in our cohort of wheelchair athletes on their physical performance and health, preventative strategies should focus on continued education to reduce IC-related complications. Future studies should aim to determine predictors for complications associated with IC and evaluate the success of continued education and preventive strategies in this target population.

## Supplementary information

Supplementary file 1 - Study questionnaire

## Data Availability

The datasets generated and/or analysed during the current study are available from the corresponding author on reasonable request.
