# Supplementary material for: Prevalence of self-reported complications associated with intermittent catheterization in wheelchair athletes with spinal cord injury
Source: Spinal Cord. 2020 Oct 13;59(9):1018–25. doi: 10.1038/s41393-020-00565-6 (PMC8397616; doi:10.1038/s41393-020-00565-6)
Supplement: Supplementary file 1 — Study questionnaire [file 41393_2020_565_MOESM1_ESM.pdf]

Supplementary file 1 - Study questionnaire

Supplementary file legend:
The study questionnaire includes 30 multiple choices questions and comprises four categories: a) demographics and SCI severity, b) questions from the ISAFSCI ‘Autonomic Standards Assessment Form’, c) history of catheterization techniques and associated complications, and d) history of IC-related inflammation / infection to genital organs associated in male individuals.

Authors:
Matthias Walter MD, PhD, Ian Ruiz, MD, Jordan W. Squair, PhD, Luis A. S. Rios, MD, Marcio A. Averbeck, MD, and Andrei V. Krassioukov, MD, PhD

Title:
Prevalence of self-reported complications associated with intermittent catheterization in wheelchair athletes with spinal cord injury

Journal:
Spinal Cord (published in 2020)

Urethral injury and bladder function questionnaire

Participant – ID: ______________________________

Date of completion:

Y	Y	Y	Y	M	M	D	D

A. Demographics

1. Gender: Female / Male
2. Date of Birth: Year:_______ Month:______ Day:________
3. Date of Injury: Year:_______ Month:______ Day:________
4. Level of spinal cord injury (SCI), e.g. C6, T5, etc.: _____________
5. If you know your severity, i.e. completeness of the motor function of your SCI, please check one:

   ☐ Complete ☐ Incomplete

6. If you know your American Spinal Injury Association Impairment Scale (AIS) grade, please check one:

   ☐ A ☐ B ☐ C ☐ D

B. Questions from the International standards to document remaining autonomic function after spinal cord injury (ISAFSCI)

1. Awareness of need to empty the bladder

   ☐ Normal function
   ☐ Reduced or altered (neurological) function
   ☐ Complete loss of control
   ☐ Unable to assess due to preexisting or concomitant problems

2. Ability to prevent the leakage (continence)

   ☐ Normal function
   ☐ Reduced or altered (neurological) function
   ☐ Complete loss of control
   ☐ Unable to assess due to preexisting or concomitant problems

3. Bladder emptying method(s) (tick all that apply)

   ☐ Spontaneous voiding
   ☐ Foley catheter, urethral
   ☐ Foley catheter, suprapubic
   ☐ Self-catheterization, urethral
   ☐ Self-catheterization, stoma
   ☐ Crede maneuver
   ☐ Other, please specify: ____

C. <u>Catheterization and complications</u>

1. Do you have a suprapubic catheter put in place as a result of injury to the urethra?

   ☐ No ☐ Yes

2. Have you ever practiced urethral self-catheterization?

   ☐ No ☐ Yes

   *If "**No**", please proceed to the next section in case you are male, otherwise you have finished this questionnaire.*
   *If "**Yes**, please proceed to question #3 and continue the questionnaire with respect to your experiences with urethral self-catheterization.*

3. Specify for how long you have practiced urethral self-catheterization:__________years

4. Specify your average number of catheterizations per day:________________times/day

5. What type of catheter do you mainly use for self-catheterization?

   ☐ Hydrophilic catheter
   ☐ Non-hydrophilic PVC catheter
   ☐ Non-hydrophilic Silicon catheter
   ☐ Non-hydrophilic Red rubber (latex) catheter
   ☐ Other, please specify: ____

6. What size of catheter do you use most of the time?

   ☐ ***French sizes***, please specify: ____

7. What type of the Catheter do you use most of the time?

   ☐ Catheter with straight tip
   ☐ Catheter with bent /curved tip (such as a Tiemann catheter)
   ☐ Other, please specify: ____

8. Do you use any lubricant on the catheter?

   ☐ No ☐ Yes

9. Do you re-use your catheter?

   ☐ No ☐ Yes

10. Please specify the number of urinary tract infections you have had during the past 12 months:______________

11. Please specify the number of **antibiotic treated** urinary tract infections during the past 12 months:___________

12. Do you currently use any medications to aid in your bladder management?

☐ No ☐ Yes

*If "**No**", please proceed to **question 13**.*

Please specify which medications (tick all that apply):

☐ Botox
☐ Anticholinergics (e.g. Ditropan, Detrol, Vesicare, Trospium, Toviaz, etc)
☐ Preventative use of antibiotics
☐ Other, please specify: ____

13. Have you had any **urethral injury(ies)** as a result of catheterization **after** your SCI?

☐ No ☐ Yes

*If "**No**", please proceed to **question 16**.*

14. Have you had any **urethral injury(ies)** as a result of self-catheterization?

☐ No ☐ Yes

*If "**No**", please proceed to **question 15**.*

During which period(s) did you experience urethral injury(ies) due to self-catheterization (tick all that apply)?

☐ First year after SCI
☐ 2-5 years after SCI
☐ 6-10 years after SCI
☐ More than 10 years after SCI

15. Have you had any **urethral injury(ies)** as a result of catheterization by medical personnel?

☐ No ☐ Yes

*If "**No**", please proceed to **question 16**.*

During which period(s) did you experience urethral injury(ies) due to catheterization by medical personnel (tick all that apply)?

☐ First year after SCI
☐ 2-5 years after SCI
☐ 6-10 years after SCI
☐ More than 10 years after SCI

16. Do you sometimes experience pain in the urethra during catheterization?

☐ No ☐ Yes

*If "**No**", please proceed to **question 17**.*

How often do you experience pain in the urethra during catheterization?

☐ Every catheterization
☐ One or more times every week
☐ One or more times every month
☐ One or more times every year
☐ Less than yearly

17. Do you sometimes experience difficulties inserting the catheter into the urethra?

☐ No ☐ Yes

*If "**No**", please proceed to **question 18**.*

How often do you experience difficulties inserting the catheter?

☐ Every catheterization
☐ One or more times every week
☐ One or more times every month
☐ One or more times every year
☐ Less than yearly

18. Do you sometimes notice blood on the catheter after withdrawal from the urethra?

☐ No ☐ Yes

*If "**No**", please proceed to the next section in case you are male, otherwise you have finished this questionnaire.*

How often do you notice blood on the catheter after withdrawal?

☐ Every catheterization
☐ One or more times every week
☐ One or more times every month
☐ One or more times every year
☐ Less than yearly

D. <u>The remaining questions are only applicable to male respondents</u>

1. Have you ever experienced any inflammation in the testicles?

☐ No ☐ Yes

*If "**No**", please proceed to **question 2**.*

During which period(s) did you experience inflammation in the testicles (tick all that apply)?

☐ First year after SCI
☐ 2-5 years after SCI
☐ 6-10 years after SCI
☐ More than 10 years after SCI

2. Have you ever experienced any inflammation in the epididymis?

☐ No ☐ Yes

*If "**No**", please proceed to **question 3**.*

During which period(s) did you experience inflammation in the epididymis (tick all that apply)?

☐ First year after SCI
☐ 2-5 years after SCI
☐ 6-10 years after SCI
☐ More than 10 years after SCI

3. Have you ever experienced any inflammation in the prostate?

☐ No ☐ Yes

*If "**No**", you have finished the questionnaire.*

During which period(s) did you experience inflammation in the prostate (tick all that apply)?

☐ First year after SCI
☐ 2-5 years after SCI
☐ 6-10 years after SCI
☐ More than 10 years after SCI

Thank you for taking your time to answer this questionnaire!
